# Improvement of Myofascial Lower Back Pain During Standing and Forward Bending Through Manual Physiotherapy to Increase Ankle Dorsiflexion: A Case Report

**DOI:** 10.7759/cureus.93366

**Published:** 2025-09-27

**Authors:** Hiroshi Maruoka, Yuichi Takata, Takahiro Matamura, Tomohiro Nomura, Mitsugu Takahashi

**Affiliations:** 1 Department of Rehabilitation, Faculty of Healthcare and Science, Graduate School of Rehabilitation Sciences, Hokkaido Bunkyo University, Eniwa, JPN; 2 Department of Prosthetics and Orthotics, Hokkaido University of Science, Hokkaido, JPN; 3 Department of Orthopedics, Takahashi Orthopaedics Clinic, Chitose, JPN

**Keywords:** ankle dorsiflexion, compensatory movement patterns, low back pain, manual physiotherapy, range of motion

## Abstract

Lower back pain substantially affects the quality of life of affected individuals, and effective treatment remains challenging. Here, we test the hypothesis that limitations in lower limb mobility, particularly at the ankle joint, contribute to compensatory movement patterns associated with lumbar pain. An 18-year-old male, high school javelin thrower, presented with right-sided lower back pain during forward bending in the standing position. Tenderness was noted in the right quadratus lumborum muscle, and the pain was relieved by manual anterior pelvic tilt during forward bending. Although temporary relief was achieved by improving hamstring flexibility and hip flexion range of motion (ROM), the effect was not sustained. Re-evaluation revealed posterior pelvic displacement during ankle plantarflexion, indicating a compensatory pattern because of restricted ankle dorsiflexion. Manual physiotherapy, including targeted stretching of the flexor hallucis longus (FHL) muscle, was administered to improve ankle dorsiflexion ROM. Following the intervention, the dorsiflexion ROM increased, and the patient reported a reduction in lower back pain during forward bending. At the seven-month follow-up, the patient remained pain-free and had returned to his previous level of athletic performance. Physiotherapy targeting the FHL muscle to improve ankle dorsiflexion ROM may be effective in reducing myofascial lower back pain during forward bending in the standing position.

## Introduction

Lower back pain is highly prevalent worldwide and affects the quality of life [1-3]. In Japan, its prevalence is approximately 38%. Because it is associated with functional and psychosocial impairment, reducing its prevalence is crucial.

Lumbar disorders are classified into the following: lumbar disk herniation, lumbar disk disease, lumbar spinal canal stenosis, lumbar intervertebral joint disease, lumbar spondylolysis, sacroiliac joint disease, and myofascial back pain, which are reported to be the second most prevalent after intervertebral joint disorders [4]. In patients with myofascial lower back pain, tenderness is observed in the paravertebral muscle and other lumbar muscles. It is difficult to diagnose myofascial low back pain on the basis of physical examination findings alone, and other causes, such as intervertebral joint syndrome and discogenic lower back pain, should be excluded.

Lower back pain is classified into five types according to the Movement System Impairment diagnostic system: flexion, extension, rotation, rotation with flexion, and rotation with extension [5]. Forward bending while standing is more restricted in patients with lower back pain than in those without [6]. One method of assessing flexion-type lower back pain is the lumbar pelvic rhythm (LPR) [7]. LPR shows the movement of lumbar flexion and anterior pelvic tilt in the sagittal plane during forward bending while standing. If the hamstrings are shortened in the upright forward bending position, pelvic anteversion is restricted, lumbar flexion increases, and lumbar tissue damage occurs.

Pain factors during forward bending while standing include decreased extensibility of hamstrings, decreased deep trunk muscle strength, decreased hip flexor strength, and limited hip flexion range of motion (ROM) [8]. It has been reported that improvement of hip flexor ROM and hamstring extensibility reduces lower back pain during forward bending while standing [9]. Humans control their posture by projecting the center of gravity of the body in the basal plane of plantar support while standing using hip and ankle strategies; however, the method of postural control differs from person to person. Similarly, healthy individuals use various pattern strategies for forward bending while standing. Therefore, evaluation and intervention of not only the joints adjacent to the lumbar region but also the feet during forward bending while standing can be a clue for treatment. Vadivelan et al. [10] and Yoon and Park [11] reported that the curve of the spinal column changed and lower back pain was reduced in participants during forward bending while standing after triceps stretching and ankle joint mobilization. In addition, ankle joint ROM affects standing balance.

Herein, we report the case of a patient who complained of lower back pain during forward bending while standing, and the pain was relieved following improvement of ankle dorsiflexion ROM.

## Case presentation

Patient characteristics

The case involved an 18-year-old male javelin thrower (height, 164 cm; weight, 75 kg). His first episode of back pain occurred during a track meeting in 2018. He was diagnosed with myofascial lower back pain because of tenderness in the erector spinae muscle without abnormal radiographic findings. In 2021, the patient developed right lumbar pain while practicing and was diagnosed with myofascial back pain due to mild instability between the fourth and fifth lumbar vertebrae. Radiography showed tenderness in the L3/L4, L4/L5, and L5/S1 interspinous ligaments and erector spinae muscles. Myofascial release injections were administered to the outer edge of the right quadratus lumborum muscle, and his lower back pain improved. The back pain recurred in the same area four months later during trunk rotation while participating in a track and field meet, and tenderness was observed in the L4/L5 interspinous ligaments. A diagnosis of lumbar disc disease was made, and physical therapy was started.

**Figure 1:**
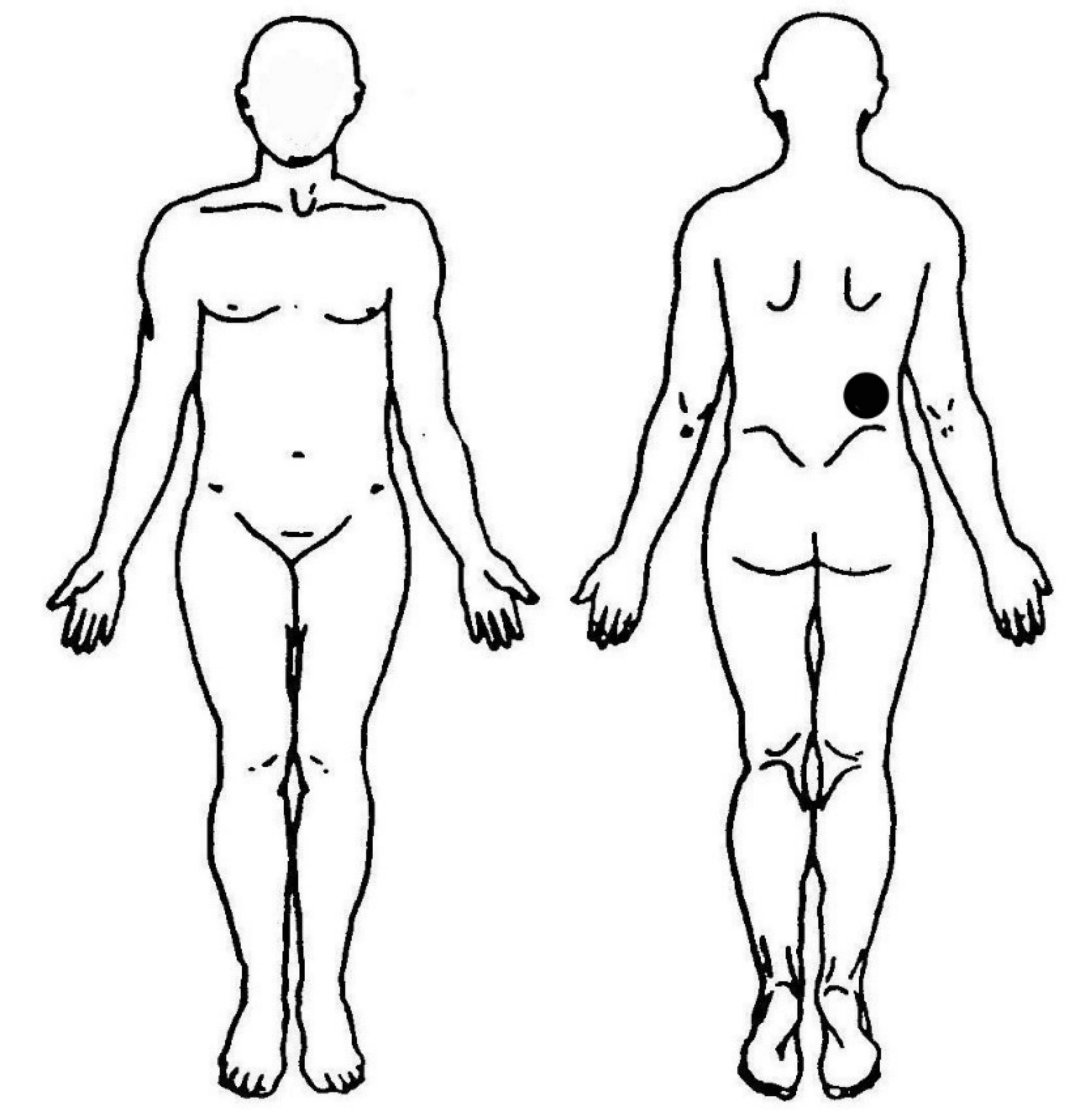
Body chart showing lower back pain during forward bending while standing The right upper iliac crest is indicated by one finger. The figure was created by the author using Adobe Illustrator software.

The chief complaint was pain in the right lumbar region that was observed while standing forward, bending, and picking up objects from the floor. The pain site was identified with one finger pointing to the upper iliac crest, where the quadratus lumborum muscle terminated (Figure 1). To prevent pain, the patient was instructed to squat down while picking up objects from the floor.

The Japanese Orthopedic Association Back Pain Evaluation Questionnaire (JOABPEQ) was used to evaluate the standing type of the patient in this case [12]. It covers the following five domains: pain-related disability, lumbar spine dysfunction, gait dysfunction, social life dysfunction, and psychological dysfunction. The JOABPEQ scores range from 0 to 100, with higher values indicating less disability and better physical and psychological functions across the five domains. The patient was asked to answer questions at the initial visit, after the intervention for improving dorsiflexion ROM of the ankle joint, and at seven months after the intervention (Table 1).

**Table 1:** Improvement in the scores of the items of the JOABPEQ observed immediately and at 7 months after treatment. JOABPEQ, Japanese Orthopedic Association Back Pain Evaluation Questionnaire; VAS, visual analog scale

	Before treatment	After treatment	After treatment (7 months)
Pain-related disorders assessed by the patient	43	100	100
Lumbar spine dysfunction assessed by the patient	75	100	100
Gait disturbance assessed by the patient	86	100	100
Social life disturbance assessed by the patient	57	100	100
Psychological disorder assessed by the patient	83	100	100
VAS	75 mm	0.6 mm	0 mm

The patient had a history of right medial hamstring avulsion and ankle sprain. No other medical or traumatic injuries were reported.

Ethical approval and informed consent

In accordance with the principles of the Declaration of Helsinki, we gave due consideration to the handling of personal information and obtained written informed consent from the patient for the publication of this case report and accompanying images. The Hokkaido Bunkyo University Institutional Ethics Committee approved the study.

Physical examination

**Figure 2:**
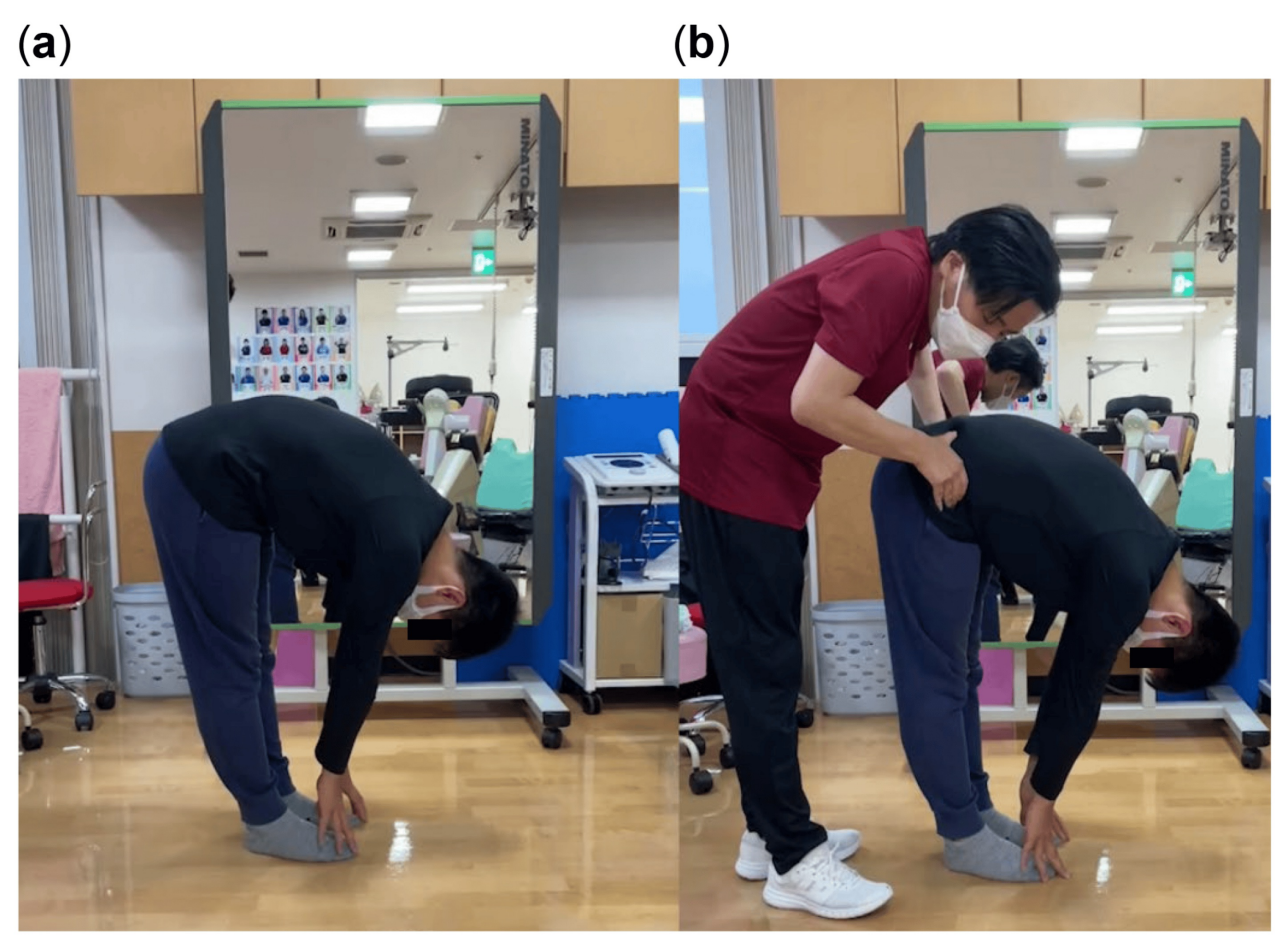
Automatic and manually assisted forward bending while standing with an anterior pelvic tilt (A) The patient was in an automatic forward bending posture while standing and complained of lower back pain on the right side (VAS score, 75 mm). The ankle joint was in plantar flexion, and the pelvis was positioned posteriorly in relation to the ankle joint. (B) The examiner manually assisted the patient with an anterior pelvic tilt during forward bending while standing. There were no complaints of lower back pain (VAS score, 0 mm). VAS, visual analog scale

The patient had no complaints of lower back pain while forward bending in the sitting position during the automatic motor test. He complained of pain in the right lumbar region during forward bending and left lateral flexion of the trunk (Figure 2A). Manual anterior pelvic tilting during forward bending while standing was found to reduce the pain (Figure 2B). Tenderness was observed in the right quadratus lumborum. No tenderness was noted in the interspinous ligament at the L4/L5 level. In the upright posture, head anteversion, thoracic kyphosis, lumbar kyphosis, and pelvic tilt were three lateral digits lower in the anterior superior iliac spine (ASIS) than in the posterior superior iliac spine (PSIS).

The ROM was 105°/110° for hip flexion, -5°/0° for knee extension dorsiflexion, 10°/15° for knee flexion dorsiflexion, 60°/60° for straight leg raise (SLR), 55°/55° for ankle plantar flexion toe metatarsophalangeal (MTP) joint extension, 20°/35° for ankle mid position MTP joint extension, and 10°/20° for MTP fixed toe joint extension (Table 2). ROM measurements were taken by an evaluator who was blinded to the intervention. The flexor hallucis longus (FHL) muscle length test described by James et al. [13] was also performed. The patient was placed in three positions: interphalangeal plantar flexion in the antigravity position, ankle dorsiflexion, and metatarsal head fixation in the ankle dorsiflexion position. The metatarsal head was extended. A positive result was defined as an angle of less than 20° or discomfort at the MTP joint of the big toe. The right big toe extension angle was 10°.

**Table 2:** Increase in the ROM for the toe MTP joint extension angle, ankle dorsiflexion angle, and SLR immediately and at seven months after treatment. MTP, metatarsophalangeal; R/L, right/left; ROM, range of motion; SLR, straight leg raise

	Before treatment (R/L)	After treatment (R/L)	After treatment (7 months) (R/L)
SLR	60°/60°	65°/65°	75°/75°
Hip flexion	105°/110°	105°/110°	110°/110°
Ankle dorsiflexion (knee joint in extension)	-5°/0°	0°/0°	10°/10°
Ankle dorsiflexion (articular knee flexion position)	10°/15°	15°/15°	20°/15°
Extension of the MTP joint of the big toe (plantar flexion position of the ankle joint)	55°/55°	55°/55°	55°/55°
Extension of the MTP joint of the big toe (mid-ankle position)	20°/35°	35°/35°	35°/35°
Metatarsal MTP joint extension (metatarsal head fixation)	10°/20°	25°/20°	30°/20°

**Figure 3:**
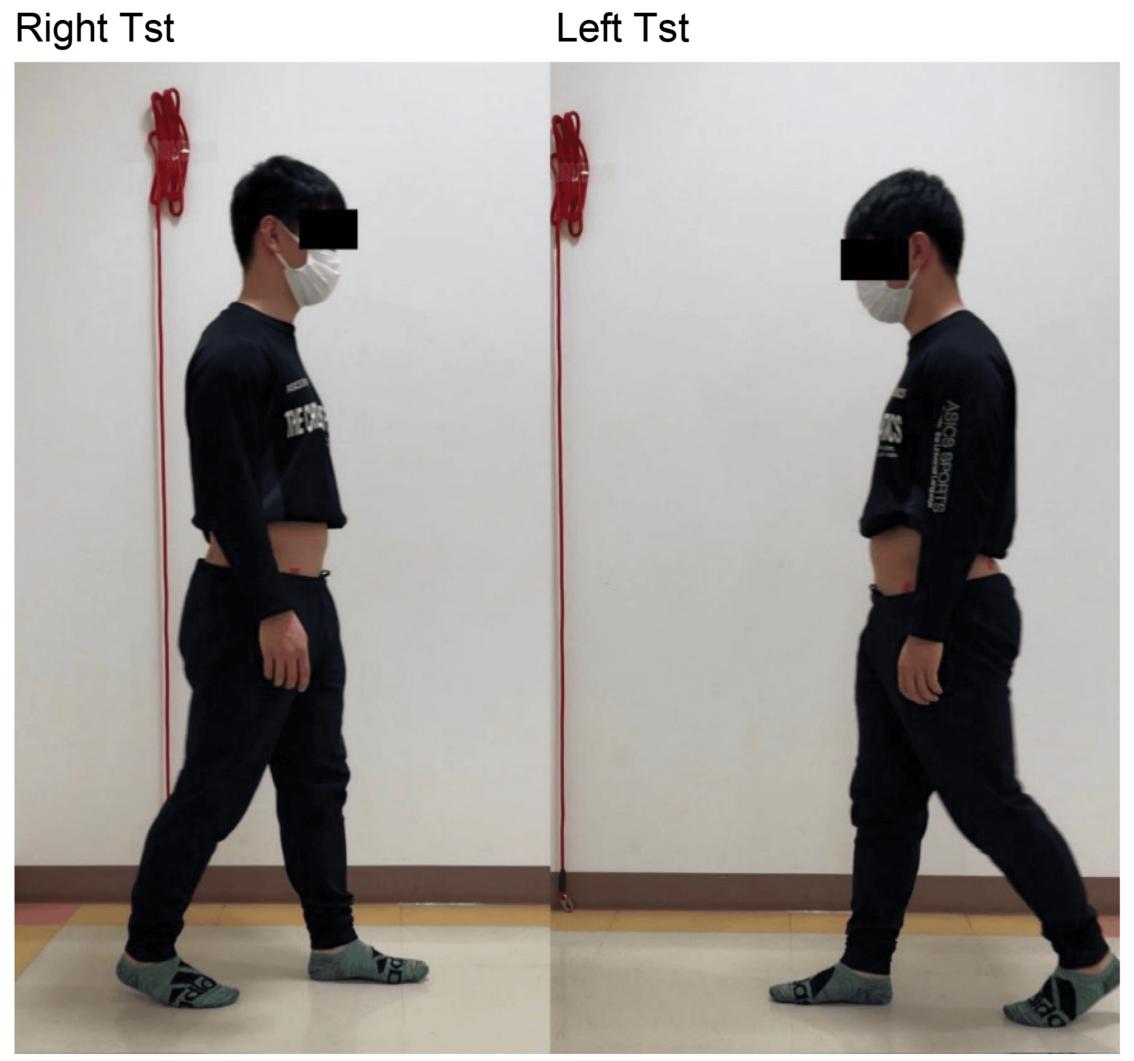
Pelvic anteversion angle at the left and right TSt Gait was observed using markers on the bilateral ASIS and PSIS. The anterior pelvic tilt angle was smaller for the right TSt than for the left TSt. ASIS, anterior superior iliac spine; PSIS, posterior superior iliac spine; TSt, terminal stance

The manual muscle test is a hip flexor strength test for both right and left hip flexors. While walking, the anteversion angle of the right pelvis in the right terminal stance (TSt) decreased relative to that of the left pelvis (Figure 3). In addition, the right foot was more abducted than the left foot, and kicking with a big toe was not observed during pre-swing. When the patient was verbally instructed to correct the abduction of the foot angle during gait, he complained of limited dorsiflexion of the right MTP joint. To examine lumbar intersegmental instability, the patient was placed in the lateral recumbent position, and the hip joint was moved in the flexion and extension directions while palpating the spinous processes. Hypermobility was observed in L4/L5 and L5/S1.

The active SLR test for lumbopelvic girdle instability testing [14] was used to test for lumbopelvic girdle instability. Bilateral twisting of the lumbar pelvic girdle was observed, and the patient had difficulty raising the lower limbs. The patient stated that it was easier to raise the lower limbs by immobilizing the pelvic girdle. This information suggests that the stability of the muscles surrounding the pelvic girdle decreased. The sciatic nerve stretch test performed with SLR yielded a negative result.

Clinical impression

The patient complained of right lumbar pain during forward bending while standing. The pain was alleviated when the pelvis was passively tilted anteriorly during the motion. The painful area was localized to the right quadratus lumborum muscle, and tenderness was noted in the same region. No tenderness was found in the L4/L5 interspinous ligament on examination, and the sciatic nerve stretch test was negative. These findings suggest that the pain was caused by the excessive elongation of the quadratus lumborum muscle because of the insufficient anterior tilting of the right pelvis during forward bending in the standing position. It has been reported that the anterior tilt of the pelvis during forward bending while standing is limited by the shortening of the hamstrings. In the present case, the pain during forward bending was reduced after stretching the hamstrings, but it flared up again later, and no long-term effect was observed. Therefore, the patient was re-evaluated by checking body movements during the operation.

Forward bending movements with standing cause backward translational movement of the pelvis [15]. The sciatic tuberosity also moves backward as the pelvis translates backward, and the distances between the origins and terminations of the hamstrings increase independently. In the present case, the ankle joint was in plantar flexion during forward bending while standing, which moved the pelvis posteriorly and limited anteversion.

Treatment

**Figure 4:**
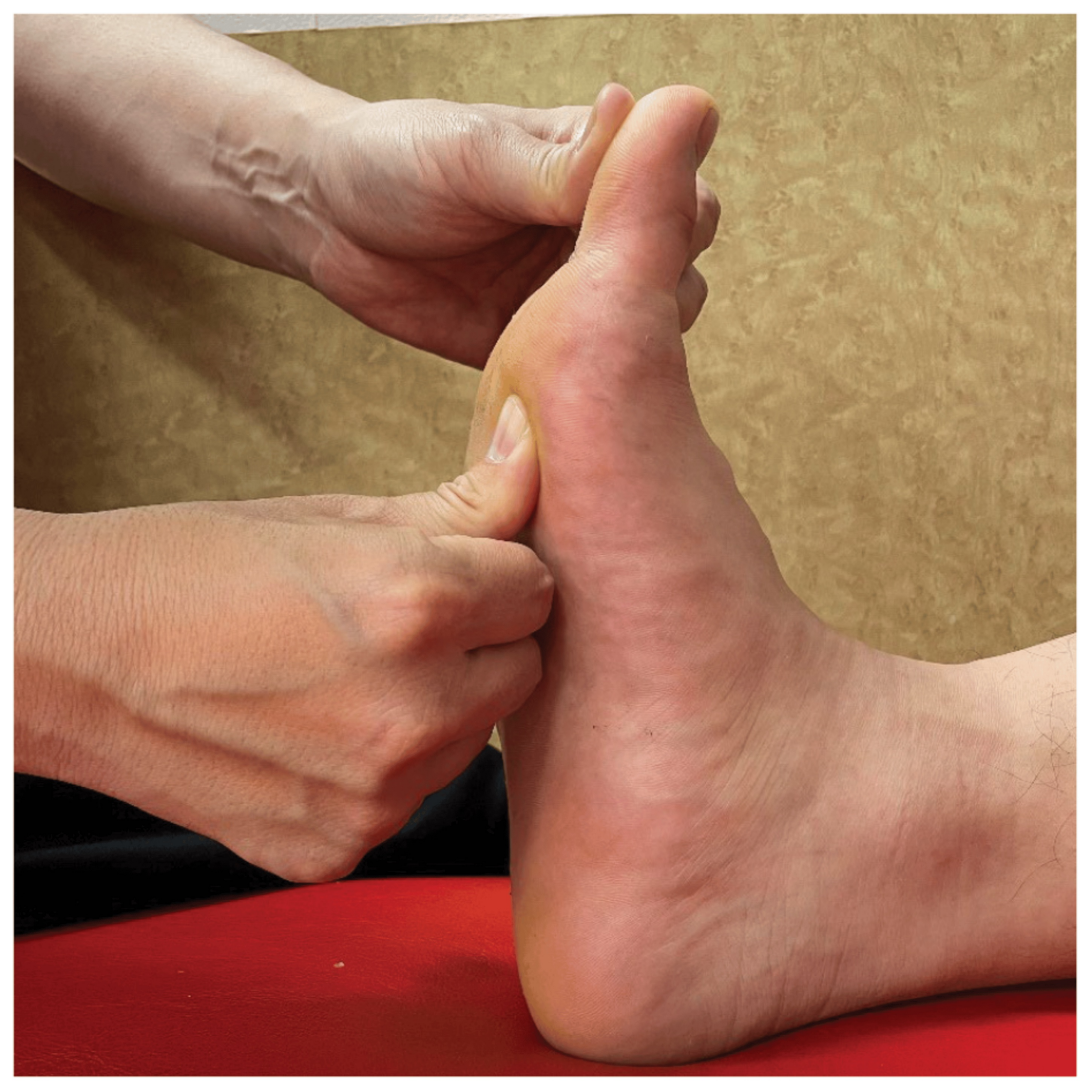
FHL stretch During FHL stretching, a towel was placed under the knee joint, which was then placed in a slightly flexed position. The ankle joint was dorsiflexed to the maximum extent. Thereafter, the intermetatarsophalangeal joint of the big toe was extended to the final range of motion while the metatarsal head of the big toe was held down, and FHL was stretched continuously. During this stretching, care was taken to fixate the metatarsal head of the big toe so that windlass action would not occur. FHL, flexor hallucis longus

To improve ankle dorsiflexion ROM, the patient grasped the distal phalanx of the big toe and extended it maximally to stretch the FHL in the maximum dorsiflexion position (Figure 4). Subsequently, the patient was placed in the maximum dorsiflexed position of the ankle joint, and the talar bone was grasped for dorsal mobilization to the talocrural joint in the grade III position [16]. Additionally, the patient was placed in knee extension, the calcaneus was grasped, and the ankle joint was dorsiflexed dynamically to stretch the gastrocnemius muscle.

Home exercise

The FHL was trained through home exercises, including hamstring self-stretching, abdominal bracing, and hip flexion exercises. The patient was instructed to perform the home exercises daily at 5-6 p.m.; the order was hamstring self-stretching followed by abdominal bracing and hip flexion exercises. After muscle stretching, abdominal bracing and hip flexion exercises were performed 20 times each.

Outcomes

**Figure 5:**
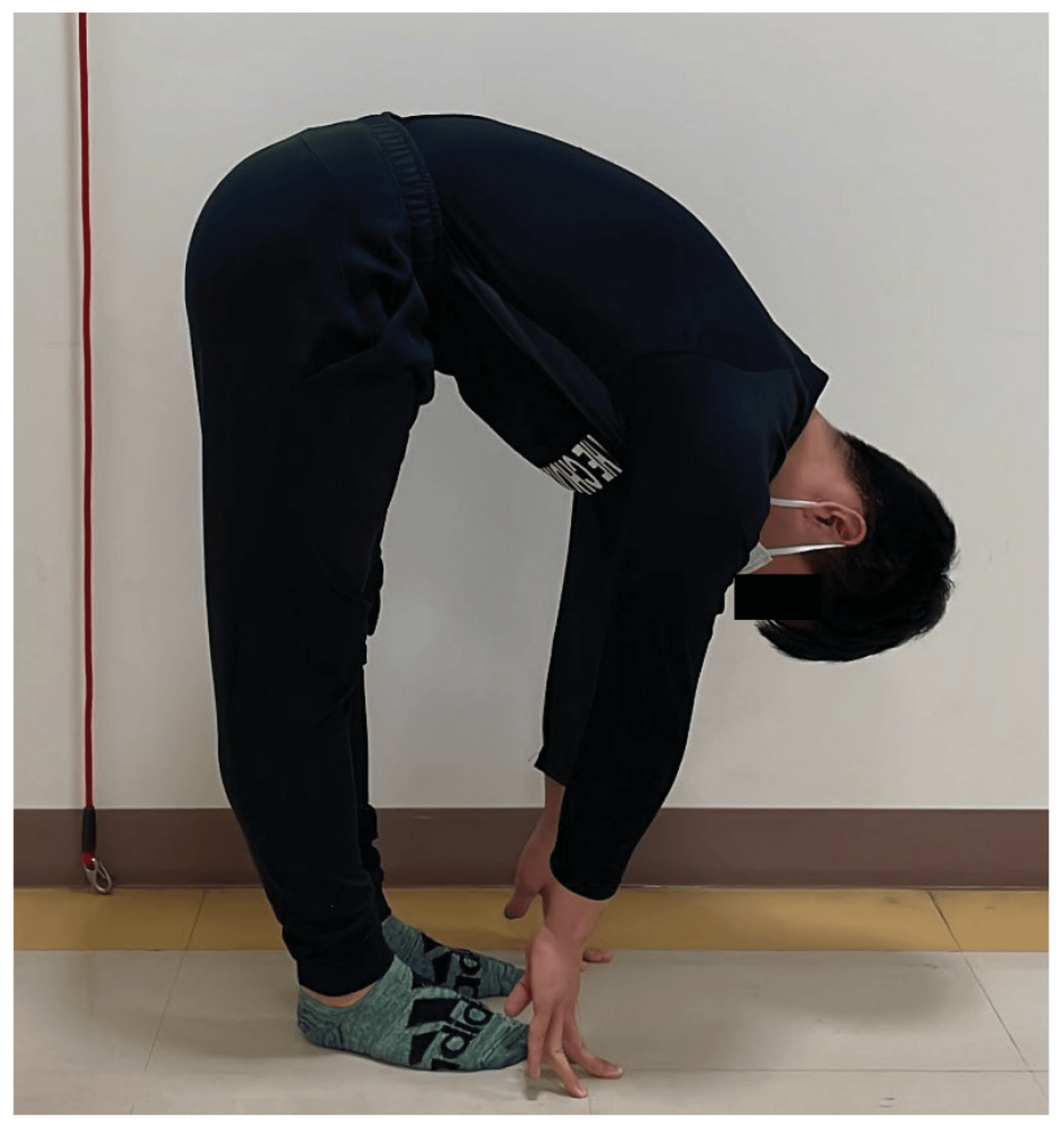
Forward bending while standing at seven months after the initiation of physical therapy No right lower back pain was reported during forward bending while standing seven months after the ankle dorsiflexion intervention (VAS score, 0 mm). Compared with the image in Figure 2A, the ankle joint was in dorsiflexion during forward bending while standing, and the pelvis was anterior to the ankle joint. VAS, visual analog scale

After intervention for ankle dorsiflexion ROM, the visual analog scale (VAS) score for standing forward flexion ranged from 75 to 0.6 mm. Reassessment performed seven months later revealed a VAS score of 0 mm (Table 1). As a result, lower back pain during forward bending while standing improved (Figure 5). The ROM was 0°/0° for ankle dorsiflexion during knee extension, 15°/15° for ankle dorsiflexion during knee flexion, and 25° for the FHL muscle length test. The hip flexion manual muscle test score was 5 for both the right and left hip joints, and no lumbopelvic girdle torsion was observed in the active SLR test. During walking, the patient no longer experienced tightness in the dorsal aspect of the right big toe MTP joint during right TSt. The JOABPEQ score for all items reached 100 after the ankle dorsiflexion ROM intervention. The reassessment at seven months showed that all items remained satisfactory.

## Discussion

In the present case, right lumbar pain during forward bending while standing was treated with stretching of the right FHL, dorsal talocrural joint mobilization, and home exercises. Improvement was observed after seven months.

The VAS score of the patient’s right lumbar pain during forward bending while standing was 75 mm. This decreased to 0.6 mm after the ankle dorsiflexion ROM intervention and 0 mm after seven months.

In addition to the anterior pelvic tilt and hip flexion, the pelvis has been reported to translate backward relative to the feet during forward bending while standing [15]. This pelvic movement translates backward when the ankle joint is in a more plantarflexed position. Therefore, when the pelvis translates more posteriorly during forward bending while standing, the distance between the origin and termination of the hamstrings increases. Consequently, the hamstrings become more extended, which limits pelvic anteversion during forward bending while standing and increases the extension stress on the lumbar square muscle.

Sagittal movements of the spine, pelvis, and hip joints have been reported in relation to forward bending while standing, but the only studies on sagittal movements of the ankle joint were conducted by Vadivelan et al. [10] and Yoon and Park [11]. They reported that the curve of the spinal column changed after triceps stretching and ankle joint mobilization, and back pain during forward bending while standing was reduced. However, the details of the tissues causing back pain and the mechanism by which the pain improved were not discussed. In the present study, an improvement in ankle dorsiflexion ROM contributed to the reduction of lower back pain during forward bending in the standing position.

The cause of ankle dorsiflexion ROM limitation in our case was thought to be a history of medial sprain of the right ankle joint. Dorsiflexion ROM limitation of the ankle joint is associated with FHL [17], decreased extensibility of the triceps femoris muscle, decreased gliding of the Kager’s fat pad [18], and decreased intra-articular motion of the talocrural and tibiofemoral joints [19]. The interviews showed that the patient had been walking with the ankle in the abduction position to prevent pain after an ankle joint internal rotation sprain. While walking, the toes extend from the TSt to the pre-swing and a forefoot rocker occurs. However, we believe that daily walking with the foot maintained in an abducted position after an ankle joint internal rotation sprain may have altered the gait pattern in such a way that extension of the first MTP joint during TSt was restricted. This restriction likely contributed to decreased extensibility of the FHL, which, in turn, may have led to a limitation of ankle dorsiflexion. As a result, the ankle joints became plantarflexed during forward bending while standing, and the pelvis moved backward, leading to lower back pain.

In the present study, we focused on the entire motion of the lumbar region and not just the adjacent joints on the basis of the history of lower back pain during forward bending while standing. We believe that this facilitated the resolution of the lower back pain and longer-term effects.

The JOABPEQ assesses a treatment as “effective” when one of the following conditions is met: the post-treatment score is higher than the pre-treatment score by 20 points or more, the pre-treatment score is less than 90 points, and the post-treatment score is higher than the pre-treatment score by 90 points or more [20]. All scores on the JOABPEQ after intervention for ankle dorsiflexion ROM were satisfactory, thus suggesting that the intervention to improve ankle dorsiflexion ROM was effective for lumbar pain during forward bending. Improvement was observed in not only pain-related disability and lumbar spine dysfunction but also social life and psychological disabilities. The lowest score before the intervention was for social disability, and the largest increase was observed after the intervention. This was evaluated using the following items: Q4-1, “Owing to back pain, you do not do your usual work at home?” and Q4-2, “Have you ever been unable to do your work or usual life activities as much as you expected because of your poor physical condition?” Our patient was a student, and “work” may refer to his school life. During the interview, the patient complained of lower back pain when picking up things from the floor and mentioned that he crouched down to pick up things to prevent pain. The pain during forward bending while standing improved, and he could pick up objects in the same manner as before. In addition, the improvement in back pain enabled him to compete in club activities without pain. Therefore, we believe that this improvement was reflected in the improved social disability score.

Psychosocial factors have been reported to influence lower back pain [20]. Therefore, we considered that the improvement in lower back pain by physical therapy also improved the psychological disability scores.

A limitation of the study is that for the evaluation of the FHL, only ankle MTP joint dorsiflexion and fixed toe MTP joint dorsiflexion ROM were assessed. We did not directly evaluate FHL changes, and ultrasound device measurements should be performed in future studies for a more detailed evaluation.

## Conclusions

This case report demonstrates that improvement in ankle dorsiflexion ROM played a crucial role in reducing lower back pain during forward bending in the standing position. Manual physical therapy including stretching of the FHL increased ankle dorsiflexion ROM, resulting in more anterior pelvic positioning during forward bending, compared with that pre-intervention. Consequently, the reduction in hamstring elongation stress and enhanced pelvic anterior tilt contributed to pain reduction during forward bending in the standing position. Moreover, this therapeutic effect was sustained over the long term.

The findings from this case suggest that assessment and intervention aimed at increasing ankle dorsiflexion ROM may be effective for reducing lower back pain during forward bending in the standing position. However, this case report has some limitations. It is based on a single-subject design, which limits the generalizability of the findings, and the length of the FHL muscle was not assessed using standardized or imaging-based measurements. Therefore, larger cohort, controlled studies are warranted to further investigate the effect of ankle ROM improvement on lower back pain.
